# Intensive Treatment with Ultrasound Visual Feedback for Speech Sound Errors in Childhood Apraxia

**DOI:** 10.3389/fnhum.2016.00440

**Published:** 2016-08-30

**Authors:** Jonathan L. Preston, Megan C. Leece, Edwin Maas

**Affiliations:** ^1^Department of Communication Sciences and Disorders, Syracuse UniversitySyracuse, NY, USA; ^2^Haskins LaboratoriesNew Haven, CT, USA; ^3^Department of Communication Sciences and Disorders, Temple UniversityPhiladelphia, PA, USA

**Keywords:** childhood apraxia of speech, ultrasound, visual feedback, intensive treatment program, speech therapy

## Abstract

Ultrasound imaging is an adjunct to traditional speech therapy that has shown to be beneficial in the remediation of speech sound errors. Ultrasound biofeedback can be utilized during therapy to provide clients with additional knowledge about their tongue shapes when attempting to produce sounds that are erroneous. The additional feedback may assist children with childhood apraxia of speech (CAS) in stabilizing motor patterns, thereby facilitating more consistent and accurate productions of sounds and syllables. However, due to its specialized nature, ultrasound visual feedback is a technology that is not widely available to clients. Short-term intensive treatment programs are one option that can be utilized to expand access to ultrasound biofeedback. Schema-based motor learning theory suggests that short-term intensive treatment programs (massed practice) may assist children in acquiring more accurate motor patterns. In this case series, three participants ages 10–14 years diagnosed with CAS attended 16 h of speech therapy over a 2-week period to address residual speech sound errors. Two participants had distortions on rhotic sounds, while the third participant demonstrated lateralization of sibilant sounds. During therapy, cues were provided to assist participants in obtaining a tongue shape that facilitated a correct production of the erred sound. Additional practice without ultrasound was also included. Results suggested that all participants showed signs of acquisition of sounds in error. Generalization and retention results were mixed. One participant showed generalization and retention of sounds that were treated; one showed generalization but limited retention; and the third showed no evidence of generalization or retention. Individual characteristics that may facilitate generalization are discussed. Short-term intensive treatment programs using ultrasound biofeedback may result in the acquisition of more accurate motor patterns and improved articulation of sounds previously in error, with varying levels of generalization and retention.

## Introduction

Ultrasound imaging can provide visualization of the tongue, an important but difficult-to-see mobile articulator that is used for production of most speech sounds. By holding a transducer beneath the chin, speech-language pathologists and their patients can view a patient’s tongue movements in real time. These images can be used to explicitly cue changes to the shape and position of the tongue to address sound errors. Studies have shown that using ultrasound images as feedback can result in improved speech sound accuracy for a wide range of clients, including: adults with acquired apraxia (Preston and Leaman, 2014) or with speech impairment following glossectomy (Blyth et al., 2016) and children with residual articulation errors (Adler-Bock et al., 2007; McAllister Byun et al., 2014; Preston et al., 2014), hearing impairment (Bacsfalvi, 2010; Bacsfalvi and Bernhardt, 2011) and childhood apraxia of speech (CAS; Preston et al., 2013). Visual feedback technologies may be a useful adjunct or supplement to other therapies, particularly for individuals whose speech errors have not responded to traditional (non-technological) treatment.

### Acquisition vs. Learning of Speech Movement Patterns

The development of new speech movement patterns can be broadly summarized in two stages: acquisition and learning. Acquisition refers to the establishment of a new movement. Learning, however, may be evidenced by generalization (e.g., to untrained words or longer linguistic units) and retention over time. Acquisition precedes learning, yet the factors that facilitate acquisition are not necessarily those that facilitate learning (Maas et al., 2008). For example, ultrasound feedback provides *knowledge of performance feedback* (i.e., information about tongue movements, which is hypothesized to facilitate acquisition), rather than *knowledge of results feedback* (i.e., knowledge of whether the sound is produced correctly/incorrectly, which is hypothesized to facilitate learning).

Additionally, treatment schedules may be viewed as *massed practice* (i.e., practice spaced over a short period of time), or *distributed practice* (i.e., practice over an extended period of time). Predictions from schema-based motor learning theory suggest that knowledge of performance feedback and massed practice may be particularly beneficial for acquiring new movement patterns, but may hinder learning (Maas et al., 2008; Schmidt and Lee, 2011).

### Ultrasound Treatment and Childhood Apraxia of Speech

CAS is a neurological speech disorder in which impaired speech motor control may lead to inaccurate and inconsistent production of speech sounds, disrupted transitions between sounds, and impaired prosody (ASHA, 2007). It has been hypothesized that feed-forward motor control is impaired in CAS (Terband et al., 2009); thus, enhancing feedback may help develop stable and accurate speech motor plans (Preston et al., 2013). Ultrasound feedback of the tongue can facilitate increased accuracy on trained speech movements for some, but not all, children with CAS. For example, Preston et al. (2013) reported on six children aged 9–15 years with CAS who showed acquisition and generalization to untreated words for at least some of the trained sound sequences (e.g., /sk, ɑɹ, ɹe, kl/). However, in a follow-up study, two of three children treated for /ɹ/ showed evidence of acquisition within sessions but minimal generalization, indicating individual variation in treatment response (Preston et al., 2016b). This suggests that ultrasound has the potential to benefit at least some individuals with speech errors related to CAS.

### Intensive Speech Therapy Programs

Although access to ultrasound technology in speech therapy is currently limited, one approach to expanding access is through intensive short-term therapy programs (i.e., massed practice). There is evidence that speech therapy provided in frequent sessions multiple times per week can yield superior outcomes over traditional, less frequent service delivery (Allen, 2013; Namasivayam et al., 2015; Kaipa and Peterson, 2016), and some motor-based speech treatments are specifically designed with intensive schedules in mind (Ramig et al., 2001; Strand et al., 2006; Murray et al., 2014).

### Present Study

In this report, we explored whether an intensive 2-week therapy program including ultrasound feedback could yield measurable improvements in speech sound accuracy for school-age children with persisting speech errors associated with CAS. It was hypothesized that an intensive program using ultrasound feedback would result in successful acquisition of speech patterns that were previously in error. Schema-based motor learning theory suggests that this acquisition-focused intervention may not facilitate generalization or retention; thus, a secondary goal was to determine whether the treatment could also facilitate learning.

## Materials and Methods

### Participants

Three English-speaking children with CAS ages 10–14 years (pseudonyms Alex, Ben, Craig) who lived geographically far from the treatment site traveled to attend an intensive treatment. All had been receiving speech-language therapy since age two. Assessment and treatment sessions were conducted by certified speech-language pathologists (the first and second authors). This study was carried out in accordance with the recommendations of the Syracuse University Institutional Review Board with written informed consent from all participants. All participants gave written informed assent in accordance with the Declaration of Helsinki.

CAS diagnosis was reported by referring clinicians, and was confirmed by the authors. The diagnosis was based on inconsistent errors, prosodic impairments and difficult sequencing/transitioning between sounds and syllables (ASHA, 2007). Tasks used to evaluate these features are outlined below. Clinical judgment, based on performance across all tasks, was used to verify the presence of CAS.

### Assessments

The first morning of the program was devoted to speech assessments, with additional tasks administered on subsequent days (for scores, see Table 1).

**Table 1:** **Participant characteristics and performance on standardized and non-standardized assessments**.

	Alex	Ben	Craig
Age (years; months)	13;2	14;3	10;8
Gender	M	M	M
History of previous therapy (ages)	2 years to present	18 months to present	2 years 6 months to present
GFTA-2 Standard score	<40	54	<40
LAT Standard score	<60	<57	<61
LAT # of inconsistent words (out of 12)	3	3	6
% /ɹ/ correct 15-sentences imitated	11	0	72
% /s/ correct 15-sentences imitated	30	98	0
Multisyllabic word repetition % Consonants correct	92	85	81
Multisyllabic word repetition % Lexical stress correct	100	85	55
Inconsistency task Average number of novel productions	2.88	2.88	2.4
Maximum performance task Apraxia score	0	2	2
Maximum performance task Dysarthria score	0	0	0
Stimulability % correct (phonemes assessed)	12 (/ɹ/)	0 (/ɹ/)	0 (/s, ʧ/)
PPVT-4 Standard score	108	107	100
CELF-4 Formulated Sentences scaled score	7	8	7
CELF-4 Recalling Sentences scaled score	5	9	5
CTOPP-2 Phonological Awareness composite	103	92	75
Non-word repetition % Phonemes correct	86	93	84
WASI-2 Matrix Reasoning *T* score	45	39	37
Hearing status	Passed screening	Passed screening at	Bilateral moderate rising
	bilaterally at 20 dB	20 dB in L, failed in R	to mild hearing loss with normal
		(threshold of 30 dB at 1 and 4 kHz)	thresholds from 2–4 kHz; wore aids

*Notes: GFTA-2, Goldman-Fristoe Test of Articulation-2; LAT, Linguisystems Articulation Test; PPVT-4, Peabody Picture Vocabulary Test-4; CTOPP-2, Comprehensive Test of Phonological Processing-2; WASI, Wechsler Abbreviated Scale of Intelligence-Second Edition. Standard scores have mean of 100 and SD of 15. Scaled scores have a mean of 10 and SD of 3. T scores have a mean of 50 and SD of 10*.

#### Speech Production Measures

To confirm the presence of a speech sound disorder, the Goldman-Fristoe Test of Articulation-2 (GFTA-2; Goldman and Fristoe, 2000) and the Linguisystems Articulation Test (LAT; Bowers and Huisingh, 2011) were administered. A Multisyllabic Word Repetition Task (Preston and Edwards, 2007) required repetition of 20 challenging words of 3–6 syllables (e.g., “specificity”) to evaluate segmental and suprasegmental accuracy. A conversational sample was also collected.

A sentence imitation task, consisting of 15 sentences with late-developing phonemes, was administered to evaluate speech sound accuracy in connected speech before and after treatment (Preston et al., 2016b).

A researcher-developed Inconsistency Task required eight consecutive productions of eight multisyllabic words. Phonetic transcriptions were compared across repeated attempts. Thus, if a child produced “rectangle” four different ways in eight attempts of the word, the score for “rectangle” was four. A variability score for each word was computed and averaged.

A maximum performance task evaluated maximum duration of /ɑ/, /mɑ mɑ/, /f/, /s/, /z/, repetition rate for syllables /pʌ/, /tʌ/, /kʌ/, and rapid sequences of /pʌ tʌ kʌ/ (Thoonen et al., 1999; Rvachew et al., 2005). Apraxia scores were based on sequencing and dysarthria scores were based on maximum duration and repetition rate: 0 represented “not dysarthric/apraxic”, 1 represented “undefined” and 2 represented “dysarthric/apraxic”.

Pre-treatment stimulability of sounds in error (see Miccio, 2002) was measured through imitation in syllable-initial, syllable-final and intervocalic positions (e.g., /ɹɑ, ɑɹ, ɑɹɑ/) in 11 syllables, each repeated three times (33 tokens).

#### Oral Language

Language skills were measured for descriptive purposes. The Peabody Picture Vocabulary Test-4 (PPVT-4, Dunn and Dunn, 2007) and the Formulated Sentences and Recalling Sentences subtests of the Clinical Evaluation of Language Fundamentals-4 (Semel et al., 2003) were administered.

#### Phonological Processing

Phonological processing skills were evaluated using the Phonological Awareness Composite of the Comprehensive Test of Phonological Processing-2 (CTOPP-2, Wagner et al., 2013) and a nonword repetition task (Dollaghan and Campbell, 1998).

#### Non-Verbal Ability

The Matrix Reasoning subtest of the Wechsler Abbreviated Scales of Intelligence-2 (WASI-2; Wechsler, 2011) was administered to characterize visual perception and reasoning.

### Therapy Program Overview

**Figure 1:**
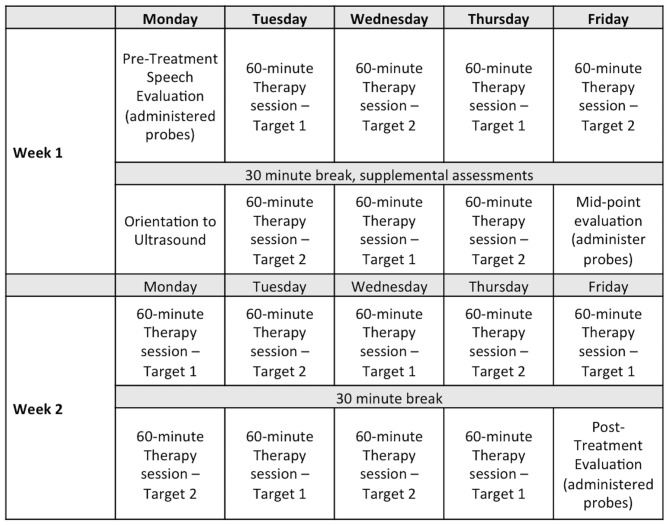
**Sample assessment and therapy schedule for a 2-week intensive treatment program.** Note: Eight 1-h sessions were provided on each of two treatment targets for a total of 16 h of therapy.

Participants attended approximately 2.5 h of therapy (or evaluations) per day from Monday to Friday for 2 weeks. Each hour of treatment addressed one phoneme in a syllable position (onset or rhyme). There were two targets per participant; thus, each target was treated for 8 h (totaling 16 h of treatment). See Figure 1 for a sample schedule.

### Data Collection

Participants wore a Sennheiser MKE-2 lapel microphone with recordings sampled at 44 kHz. Acquisition was tracked by the number of correct attempts at the targets during treatment sessions. Generalization was tracked by probe lists read by the participants on the first and fifth mornings of treatment, and following the final treatment session; additionally, participants submitted recordings of probes recorded at home (on smartphones) 2 weeks prior to the first visit to aid the researchers in target selection, and again 1–3 weeks after treatment to evaluate retention. Generalization probes assessed word-level accuracy of treated phonemes on at least 50 untrained words. For example, /ɹ/ onset was sampled in probes evaluating onset singleton (e.g., “red”), and clusters (e.g., “brown”). Recordings of probes were edited into individual audio files, randomized, and played to four trained listeners who were blind to the information of when the recordings were collected. Each word was scored by each listener as 0 (incorrect) or 1 (correct) for the perceived accuracy of the target sound. The average rating across listeners was used to evaluate progress. Fleiss’s Kappa, an estimate of reliability, was 0.642 (95% CI 0.631–0.654).

### Treatment Targets

Targets were defined as a phoneme in a syllable position and were: Alex: /ɹ/ onset, /ɹ/ rhyme; Ben: /ɹ/ onset, /ɹ/ rhyme; Craig: /s/ onset, /ʧ/ rhyme. Errors for Alex and Ben involved derhotacized distortions of /ɹ/, whereas Craig’s errors involved lateralized distortions of /s/ and /ʧ/.

### Treatment Procedures

The treatment procedures were similar to those described elsewhere (Preston et al., 2013, 2014, 2016a,b). Each hour of treatment began with 50 trials (6–10 min) of auditory perception training to facilitate perceptual awareness of speech errors. The researcher-developed perception modules consisted of authentic recordings of correct and incorrect productions of single words (cf. Rvachew, 1994) that contained the phoneme addressed in that session. Participants judged whether each token was “right” or “wrong” and received feedback after each trial. Although modules were repeated during the study, each child was exposed to at least 100 different tokens of each sound in each word position.

The remainder of each treatment hour involved production training and was divided into four 12-min Time Periods (A, B, C and D). Periods A and C included ultrasound biofeedback to facilitate a deeper understanding of the articulatory requirements of the targets. Periods B and D included practice without the ultrasound to facilitate generalization without relying on biofeedback.

Production practice was also divided into two stages: pre-practice and Structured Practice. Pre-practice involved facilitating correct forms of the targets. Verbal cues, pictures and descriptions of the articulatory requirements of the sounds were provided; these were supported during Time Periods A and C with ultrasound biofeedback. Pre-practice was loosely structured and involved phonetic cues and shaping techniques (e.g., shaping /s/ from /t/, shaping /ɹ/ from /l/ or /ɑ/) to establish correct productions. Once 12 correct renditions of the target were produced, Pre-practice ended and the remainder of the session involved Structured Practice.

During Structured Practice, chaining (Chappell, 1973; Preston et al., 2014, 2016a,b) was used to systematically progress to increasingly complex targets. Items progressed from syllables (e.g., /si/) to monosyllabic words (e.g., seed), multisyllabic words (e.g., seedling), set phrases (e.g., a seedling in the dirt) and self-generated sentences (e.g., He put the seedling in the ground). Structured Practice included blocks of six trials; at the end of each block, a decision was made to either progress to a more complex item or return to syllable level practice. If, during any block of six trials, fewer than five productions were correct, practice returned to the syllable level but targeted a slightly different phonetic environment (e.g., /so/), which could then be chained to higher levels of complexity. At each level of linguistic complexity, a pre-determined proportion of trials was assigned to the knowledge of performance and/or knowledge of results feedback by the treating clinician. Feedback frequency was systematically reduced from five (of six) trials per block at the syllable level to three trials per block in self-generated sentences. Additionally, the type of verbal feedback was systematically adapted primarily from the knowledge of performance feedback (e.g., “I didn’t see the sides of your tongue go up for the /s/”) at the syllable level to primarily knowledge of results feedback (i.e., correct or incorrect) at higher levels of linguistic complexity.

#### Use of Ultrasound

**Figure 2:**
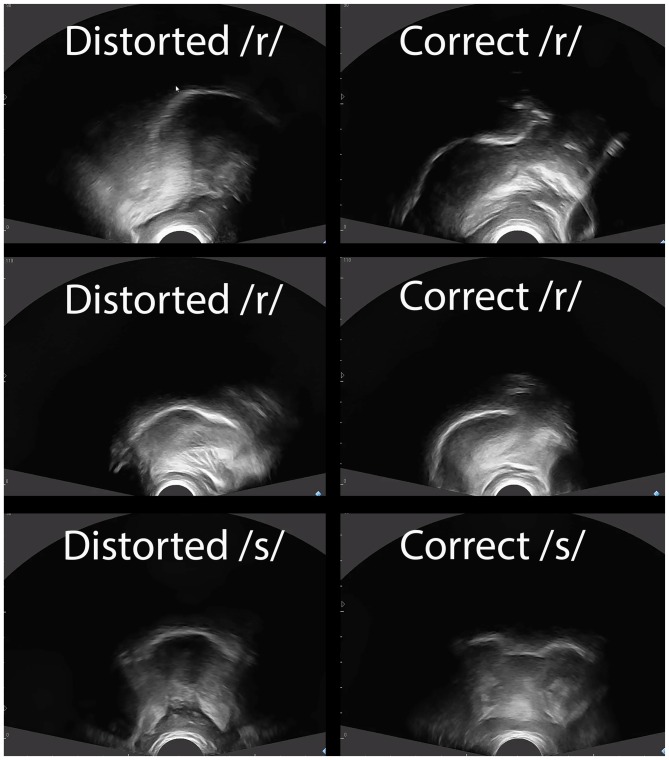
**Tongue shapes for distorted and correct productions.** Note: Sagittal views of the tongue are shown for /ɹ/ for Alex (top row) and Ben (middle row). Anterior is right and posterior is left. Note the elevation of the anterior tongue on the right side of the correct /ɹ/ productions, and the retraction of the tongue root for correct /ɹ/ relative to incorrect /ɹ/. A coronal view is shown for Craig’s /s/ (bottom row). Note that the correct /s/ has a groove in the middle of the tongue along with elevation of the lateral margins, whereas the distorted /s/ shows the sides of the tongue down.

An Echo Blaster 128 ultrasound with a PV 6.5 transducer was used during Time Periods A and C. Cues provided with the visual feedback were specific to the sounds treated and to the nature of the errors. Both sagittal and coronal views were used at the discretion of the clinician and were dependent upon the movements being cued. For Alex and Ben, whose errors primarily involved /ɹ/, a sagittal view was used to cue raising the tongue tip or blade, lowering the tongue dorsum and retracting the tongue root; a coronal view was used to cue elevation of the lateral margins and grooving at the midline of the tongue. For Craig, whose errors involved lateralized distortions of /s/ and /ʧ/, a coronal view was used exclusively. Target shapes were drawn on transparencies over the computer screen for the participants to “copy” and to provide a reference of appropriate targets. Figure 2 provides sample ultrasound images of correct and incorrect tongue shapes.

#### Treatment Fidelity

Recordings of sessions were reviewed to ensure the pre-specified type, and frequency of feedback was provided. A research assistant reviewed videos of Structured Practice from 10 randomly selected sessions. The specified verbal feedback was provided 95.5% of the time (SD: 4.4%). Additionally, inter-rater agreement between the treating clinician’s determination of correct/incorrect productions during treatment and the research assistant was 93.9% (SD: 4.3%).

## Results

**Figure 3:**
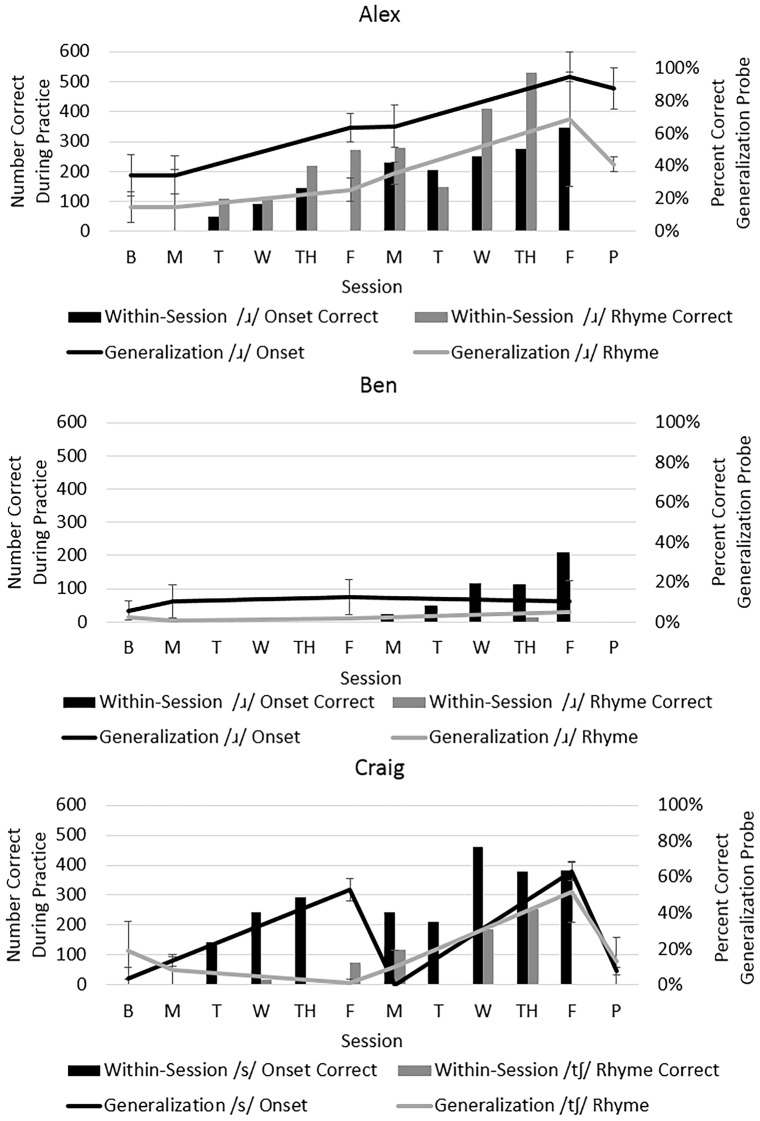
**Performance during acquisition and generalization per participant.** Note: The bars represent acquisition (number of trials correct during each session, left vertical axis); the lines represent generalization as assessed by percentage correct on probes for untreated words (right vertical axis). Probe data were obtained prior to treatment (via submitted audio recordings), on the first morning, the fifth morning, and following the final treatment session. Additionally, audio recordings of probes were submitted by Alex and Craig 1–3 weeks after treatment ended to assess retention.

Acquisition was quantified by the number of correct trials during Structured Practice (bars in Figure 3). Generalization was measured by participants’ performance on untrained word probes (lines in Figure 3) and on a sentence repetition task administered before and after treatment (difference between two listeners’ ratings was 2.83% SD: 2.0%). To quantify change, two statistics were computed: raw percent change and a standardized effect size, *d*_2_ (the change from baseline divided by the pooled standard deviation of baseline and post-treatment, which can provide interpretive guidance and comparison across studies, Beeson and Robey, 2006).

Alex showed clear evidence of acquisition of his targets in the first sessions, with an increase in accuracy of his generalization scores for /ɹ/ in onset (from 34% to 90%, *d*_2_ = 9.5) and /ɹ/ in rhyme (from 17% to 71%, *d*_2_ = 16.1). Follow-up probes 1 week later revealed retention. Similar improvements in accuracy of /ɹ/ were observed on the sentence repetition task (from 12% to 51%).

Ben did not achieve correct productions within treatment sessions until the second week of therapy, and correct productions occurred primarily for /ɹ/ in onset. There was negligible change in generalization scores both for /ɹ/ in onset (from 7 to 10%, *d*_2_ = 0.4) and in rhyme (from 2% to 5%, *d*_2_ = 1.83). Similarly, negligible improvements in /ɹ/ accuracy were observed in sentences (from 1% to 2%).

Craig achieved successful productions within treatment sessions for both /s/ in onset and /ʧ/ in rhyme, although it is apparent in Figure 3 that he achieved more correct productions of /s/ during treatment. On the generalization probes, his performance increased for /s/ in onset (from 10% to 36%, *d*_2_ = 10.5) and for /ʧ/ in rhyme (from 12% to 33%, *d*_2_ = 2.2). However, Craig did not maintain these improvements without therapy. From Friday of the first week to Monday of the second, his accuracy dropped sharply. Additionally, he did not maintain gains following the conclusion of his intensive program. Probes obtained 3 weeks after therapy showed a return to baseline levels. Data further suggest he did not generalize accurate production of sibilants to sentences (from 0% to 3.5%).

## Discussion

This case report explored an intensive treatment program for children with CAS using a motor-learning approach that included ultrasound biofeedback of the tongue. The results revealed three unique profiles from the three participants. Alex showed a steady increase in acquisition with evidence of generalization and retention. Ben showed evidence of acquisition only during the second week of treatment with minimal generalization or retention. Finally, Craig showed evidence of acquisition and generalization when generalization was probed immediately; however, his retention was limited (i.e., between the first and second week of therapy, or at a 3-week follow-up). Follow-up data were limited to home recordings, and differences in recording equipment may influence those ratings.

Individual differences in response could be attributable to a number of factors. For example, Alex showed some evidence of stimulability prior to the start of treatment (i.e., 12% accuracy on /ɹ/ in imitative syllables) whereas Ben and Craig did not (i.e., 0%). Additionally, Alex had the mildest profile, with the highest scores on percent consonants correct and lexical stress on imitation of multisyllabic words. He also had the strongest phonological processing skills (see CTOPP scores), which may aid the integration of learned motor patterns into underlying phonological representations (see Preston et al., 2016b). He also performed the highest on nonverbal visual reasoning (see WASI scores). Error type did not appear to affect outcomes as both the strongest and weakest responders had /ɹ/ distortions.

It should be noted that the approach described here emphasizes acquisition of sounds with massed practice and frequent knowledge of performance feedback. An intensive program should be followed by continued practice to ensure the skills acquired are retained and generalized; however, whether such continued practice should include ultrasound feedback remains an open question. Because the treatment program incorporates a number of elements, the key components are indeterminable. Hence, it is unclear whether the intensity of treatment, the feedback from the ultrasound, the hierarchy of structured practice and feedback, or the auditory perceptual training are the essential elements. Moreover, the approach addresses speech sound accuracy and consistency but does not inherently target prosody; for individuals whose primary difficulties are prosodic, for example, other approaches would be recommended (e.g., Murray et al., 2015).

Intensive therapy programs with visual feedback may be one option for increasing speech accuracy for some school-age children with CAS. All participants showed an increased ability to perform the desired speech movements for perceptually accurate productions during treatment, but this approach did not immediately result in generalized improvements to untrained items or to connected speech for all children. Thus, an intensive program may aid acquisition of speech sounds for individuals who were not stimulable, and it may facilitate generalization and retention for some children. In sum, the potential for observable improvement in speech sound accuracy suggests that this treatment approach warrants further investigation.

## Author Contributions

JLP was the principal investigator on the grant that funded this project. He was involved in the study design, development of the treatment protocol, data collection, data analysis and manuscript preparation. MCL was involved in data collection, data analysis and manuscript preparation. EM was involved in the design, contributed to the theoretical underpinnings, and assisted in manuscript preparation.

## Funding

This study was supported by National Institutes of Health (NIH) grant R03DC013152.

## Conflict of Interest Statement

The authors declare that the research was conducted in the absence of any commercial or financial relationships that could be construed as a potential conflict of interest.
